# Cephalometric Analysis for Gender Determination Using Maxillary Sinus Index: A Novel Dimension in Personal Identification

**DOI:** 10.1155/2017/7026796

**Published:** 2017-03-08

**Authors:** Tanya Khaitan, Arpita Kabiraj, Uday Ginjupally, Ritika Jain

**Affiliations:** ^1^Department of Dentistry, Murshidabad Medical College and Hospital, Berhampore, West Bengal 742101, India; ^2^Department of Oral Pathology & Microbiology, Index Institute of Dental Sciences, Indore, Madhya Pradesh 452016, India; ^3^Department of Oral Medicine & Radiology, Kamineni Institute of Dental Sciences, Narketpally, Andhra Pradesh 508254, India; ^4^Centre for Development Studies, Trivandrum 695011, India

## Abstract

*Purpose*. Radiography is important in forensic odontology for the identification of humans. The maxillary sinus is the largest of the paranasal sinuses and first to develop. Sinus radiography has been used for identification of skeletal remains and determination of gender. Hence, the aim and objectives of the present study were to establish a new method for gender determination using maxillary sinus index from lateral cephalometric radiographs and to establish the reliability of maxillary sinus for gender determination.* Methods*. A total of 50 adult digital lateral cephalometric radiographs (25 males and 25 females) were included in the study. The maxillary sinus analysis was performed on these radiographs using the height and width measurement tools of Sidexis XG software. Maxillary sinus index was calculated, discriminant function analysis performed, and discriminant equation derived for determination of gender.* Results*. The mean maxillary sinus height and width were found to be higher in males, whereas the maxillary sinus index was greater in females. The discriminant function analysis derived in the study was able to differentiate the sex groups with sensitivity of 68% and specificity of 76%.* Conclusions*. From the results of the present study, it may be concluded that morphometric analysis of maxillary sinus can be used as a reliable tool in gender determination.

## 1. Introduction

Personal identification is a subtle perception and often one of the most significant priorities in the investigation of criminal cases, mass disasters, and in forensic concerns. Gender determination is one of the important parameters in forensic identification. The study of anthropometric characteristics is of fundamental importance to solve problems related to such cases. Among the human bones, next to the pelvis, the skull is the most easily sexed portion of the skeleton, but the determination of the gender from the skull is not well reliable until after puberty. In cases of mass disasters, even the skull and other bones are badly blemished, however it has been reported that maxillary sinuses remain intact [1, 2].

Maxillary sinuses are two air filled spaces located in the maxillary bone. The apex of the sinuses extends into the zygomatic process occupying the zygomatic bone and the floor is formed by the alveolar process, the first, second, and third molars and the roots of canines may elevate the sinuses or may perforate their floor [3]. Sinus radiography has been used for identification of skeletal remains and determination of gender. There are various imaging modalities varying from conventional techniques such as water's view and lateral cephalogram to advanced technologies including computed tomography and cone beam computed tomography [1]. Lateral cephalogram plays a predominant role providing architectural and morphological details of the skull, thereby revealing supplementary characteristics and multiple points for comparison. Various researchers have alleged this conventional radiograph as cost effective, easily available, and reliable in providing accuracy of 80–100% [1, 3, 4].

Considering this background, the aim and objectives of the present study were to develop a new method for gender determination using maxillary sinus index from lateral cephalometric radiographs, establish the reliability of maxillary sinus for gender determination, and explore whether the height, width, and maxillary sinus index can be used for determination of gender.

## 2. Material and Methods

The study was initiated after the protocol had been approved by the Institutional Ethical Committee. A total of 50 healthy subjects belonging to both genders (25 males and 25 females) of age group 25–55 years attending the outpatient department of Oral Medicine and Radiology were selected for the study by simple random sampling. Subjects with history of facial trauma, fracture of maxillary sinus, congenital developmental abnormalities, and any maxillary sinus pathology were excluded from the study. The importance and need for the study were explained to each individual.

**Figure 1:**
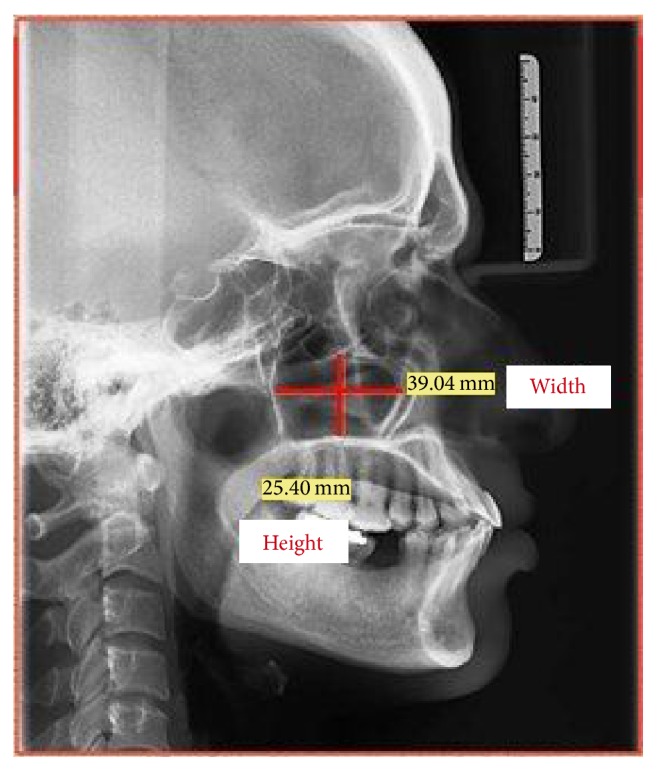
Maxillary sinus height and width measurements radiographically using Sidexis next generation software.

All the study samples were instructed to remove any dental appliances and metal objects from the head and neck region. The subjects were told about the procedure and subjected to digital lateral cephalogram (Sirona Orthophos XG Model 6229343 D3352, SN 05685) with proper radiation protection measures under exposure factors as applicable to their age. The radiographs were then stored with patients details incorporated. All radiographs were interpreted and the maxillary sinuses height and width were measured using Sidexis next generation software (version 2.5, Sirona, Germany) (Figure 1). The measurements obtained were recorded and entered in the proforma specially designed for the study. Therefore, the maxillary sinus index (MSI) was calculated as follows: MSI = maxillary sinus width/height.

The results obtained were subjected to statistical analysis using SPSS version 16.01 (statistical package for social sciences) software. The mean values and standard deviation of the maxillary sinus height, width, and MSI in males and females were obtained and tabulated using paired *t*-test. The mean differences were also calculated for the same and 95% confidence intervals (CI) evaluated. Discriminant function analysis was performed for determination of gender. Discriminant equation was also derived with gender as a classifying variable and MSI as an independent variable. The discriminant scores (*D*) thus obtained were recorded and gender differentiation was done accordingly. Sensitivity and specificity were determined to assess the accuracy of the results and reliability of the procedure. Significance level was based on *p* value < 0.05.

## 3. Results

The mean maxillary sinus height was found to be 30.4 mm in males and 28.5 mm in females and it was statistically significant with (0.5648 to 3.122) 95% CI and *p* value of 0.0066. The mean maxillary sinus width was 38 mm in males and 37.3 mm in females which was statistically nonsignificant with (−0.8230 to 2.377) 95% CI and *p* value of 0.3254. The mean MSI was higher in females (1.34) when compared with males (1.26) with (−0.1486 to −0.01389) 95% CI and a significant *p* value of 0.0202 (Table 1). In the present study the lowest value was presented by MSI, indicating MSI to be comparatively a better indicator for sex determination among all the variables.

Discriminant analysis was done using gender as a grouping variable and MSI as an independent variable and discriminant equation was obtained as follows: *D* = 11.509 − 8.871 × MSI. This equation provided to calculate “*D*” will aid in prediction of gender by substituting the values of specific measurements (MSI) in the equation. A greater calculated *D* (*D* > 0) indicates female gender, while *D* value less than the reference value (*D* < 0) indicates male gender. The more extreme the calculated *D* value from the cut-off value, the higher the probability that the predicted gender is correct (Table 2). The obtained determinant equation was applied to the study sample and revealed that 19 out of 25 were correctly identified as males and 17 out of 25 as females with sensitivity of 68% and specificity of 76% (Table 3).

## 4. Discussion

Identification of skeletal and human remains is one of the most difficult areas of expertise in forensic sciences. Determination of gender is exceptionally vital as it can positively rule out a certain percentage of possibilities instantaneously. Radiology can assist in giving precise dimensions for which certain formulae can be applied to determine gender. When the skeleton exists completely, sex can be determined with 100% accuracy. The precision rate is 98% in cases of pelvis and cranium, 95% in pelvis and long bones, and 80–90% only with long bones [3, 5]. Gender can be determined by various methodologies such as sexual dimorphism with tooth morphology, pulpal DNA analysis, study of lip prints, palatal rugae, and finger prints and even with radiological techniques by morphometric analysis of paranasal sinuses [1].

Maxillary sinus is the largest and first to develop among all the paranasal sinuses. At 10 weeks in utero, the maxillary sinus starts to develop. After birth, it continues to pneumatize into the developing alveolar ridge as the permanent teeth erupt. By 12-13 years, the sinus floor is in level with the nasal floor, and by 20 years, with the completion of the eruption of the third molars, the pneumatization of the sinus ends and it reaches 5 mm inferior to the nasal floor. Henceforth, subjects included in the present study were above the age of 20 [3, 5].

The maxillary sinus dimensions tend to stabilize after the second decade of life. Radiographic images provide adequate measurements for maxillary sinuses that cannot be approached by other means [6, 7]. Thus, the maxillary sinus height, width, and maxillary sinus index were considered in our study.

During adulthood, the shape and size of the maxillary sinus change especially due to loss of teeth. After the maximum growth period, the volume of the maxillary sinus decrease in both genders. This is attributed to the fact that the loss of minerals in the bone matrix of the entire body structure surrounding the maxillary sinus in all directions contracts the maxillary sinus and results in decrease in the maxillary sinus volume [8].

Similar study conducted by Fernandes in (2004) about gender determination from measurement of the maxillary sinuses showed that the maxillary sinus was larger in males than in females with an accuracy rate of 79.0% [9]. Teke et al. (2007) established the accuracy of gender determination of 69.4% in females and 69.2% in males [10]. Uthman et al. (2011) concluded that 74.4% of male sinuses and 73.3% of female sinuses were sexed correctly and the overall percentage for sexing maxillary sinuses correctly was 73.9% [6]. Vidya et al. (2013) studied the height, length, width, and volume of maxillary sinuses of 30 dry skulls of south Indian origin and stated that the measurements and volume of maxillary sinus of males were slightly more [11]. Chandra et al. (2014) established the accuracy and reliability of maxillary sinus in gender determination using morphometric parameters (area and perimeter), using lateral cephalogram. The correct predictive accuracy was found to be 70.8% in males and 62.5% in females [3]. Kanthem et al. (2015) found that the dimensions and volume of maxillary sinuses of right and left side using computed tomography were markedly larger in males compared with females [1]. All the results of the aforementioned studies were nearly analogous to the present study.

Gender determination is one of the integral aspects in personal identification of an unidentified cadaver, thus narrowing down the diagnosis towards an accurate possibility. Most of the bones conventionally used (pelvis, skull, long bones, etc.) are often recovered either in a fragmented, curtailed, or commingled state especially in catastrophes and mass disasters, making identification a complicated task. Various authors have reported that zygomatic bones and maxillary sinus remain intact although the skull and other bones may be badly disfigured in victims who are incinerated [1, 3].

Lateral cephalometry, being a two-dimensional conventional radiographic technique, is readily available and inexpensive and permits a good assessment of the soft tissue elements that defines the paranasal sinuses and their surrounding structures. Therefore, the morphometric analysis of maxillary sinus has been proved to be a valuable tool in the assessment of sexual dimorphism. This is relatively a new and reliable method for gender determination using maxillary sinus index. However, further studies are desirable on large sample size.

## Figures and Tables

**Table 1:** Comparison between maxillary sinus height, width, and MSI in males and females.

	Gender	*N*	Mean	Std. deviation	Std. error	95% CI	*p* value
Height	Males	25	30.4	1.87	0.37	0.5648 to 3.122	0.0066
	Females	25	28.5	2.52	0.50		
Width	Males	25	38.0	3.17	0.63	−0.8230 to 2.377	0.3254
	Females	25	37.3	3.33	0.67		
Maxillary sinus index (MSI)	Males	25	1.26	0.11	0.02	−0.1486 to −0.01389	0.0202
	Females	25	1.34	0.12	0.02		

**Table 2:** Calculation of the discriminant equation and discriminant score (*D*).

	Males	Females	
Function at group centroids	−0.375	0.375	Classified as male if *D* < 0
			Classified as female if *D* > 0

**Table 3:** Discriminant analysis showing specificity of 76% and sensitivity of 68%.

	Predicted group		Total
Original (count)			
Males	19	6	25
Females	8	17	25
%			
Males	76%	24%	100
Females	32%	68%	100
